# Simultaneous multimodal fNIRS-EEG recordings reveal new insights in neural activity during motor execution, observation, and imagery

**DOI:** 10.1038/s41598-023-31609-5

**Published:** 2023-03-29

**Authors:** Wan-Chun Su, Hadis Dashtestani, Helga O. Miguel, Emma Condy, Aaron Buckley, Soongho Park, John B. Perreault, Thien Nguyen, Selin Zeytinoglu, John Millerhagen, Nathan Fox, Amir Gandjbakhche

**Affiliations:** 1 Eunice Kennedy Shriver National Institute of Child Health and Human Development (NICHD), National Institutes of Health, Bethesda, MD USA; 2 Department of Human Development and Quantitative Methodology, University of Maryland, College Park, MD USA

**Keywords:** Biomarkers, Neurology

## Abstract

Motor execution, observation, and imagery are important skills used in motor learning and rehabilitation. The neural mechanisms underlying these cognitive-motor processes are still poorly understood. We used a simultaneous recording of functional near-infrared spectroscopy (fNIRS) and electroencephalogram (EEG) to elucidate the differences in neural activity across three conditions requiring these processes. Additionally, we used a new method called structured sparse multiset Canonical Correlation Analysis (ssmCCA) to fuse the fNIRS and EEG data and determine the brain regions of neural activity consistently detected by both modalities. Unimodal analyses revealed differentiated activation between conditions; however, the activated regions did not fully overlap across the two modalities (fNIRS: left angular gyrus, right supramarginal gyrus, as well as right superior and inferior parietal lobes; EEG: bilateral central, right frontal, and parietal). These discrepancies might be because fNIRS and EEG detect different signals. Using fused fNIRS-EEG data, we consistently found activation over the left inferior parietal lobe, superior marginal gyrus, and post-central gyrus during all three conditions, suggesting that our multimodal approach identifies a shared neural region associated with the Action Observation Network (AON). This study highlights the strengths of using the multimodal fNIRS-EEG fusion technique for studying AON. Neural researchers should consider using the multimodal approach to validate their findings.

## Introduction

Motor Execution (ME), Observation (MO), and Imagery (MI) are basic cognitive and motor skills frequently used in rehabilitation and motor learning^1^. ME and MO reflect performing an action and observing another person executing an action, respectively, whereas MI is the mental rehearsal of actions without corresponding motor output^1,2^. The Simulation Hypotheses suggest that ME, MO, and MI rely on a shared neural network (i.e., Action Observation Network, AON), and therefore, could be used alternatively/together with each other to promote learning and rehabilitation^1,3,4^. However, using unimodal neuroimaging tools, few studies have compared neural activity across all three conditions. Moreover, findings from different neuroimaging modalities are inconsistent in terms of regions showing differentiated neural activity between conditions^5,6^. Given the strengths of electroencephalography’s (EEG) temporal resolution and functional near-infrared spectroscopy’s (fNIRS) spatial resolution along with their tolerance of movement artifacts, our research group previously used a multimodal approach that includes simultaneous EEG-fNIRS recording and a fusion analytical method (i.e., structured sparse multiset canonical correlation analysis, ssmCCA) to examine neural activity over AON during ME and MO^7^. In the current study, we further examined the neural activities during MI using this approach and compared both unimodal and multimodal neural activation among the three conditions.

MI involves motor planning while inhibiting overt movements^8^. Since MI requires intense motor planning, it recruits similar neural networks (i.e., primary motor cortex, premotor cortex, supplementary motor area, anterior cingulate cortex, parietal lobule, and cerebellum) to those activated when the actual movements are executed^4^. Likewise, MO of an action elicits similar neural activity as performing that action (ME); it is posited that this helps the observer understand the goals and the intentions behind observed actions^9^. The Simulation Hypothesis suggests that ME, MO, and MI share similar neural mechanisms^3,4^. Because of these similarities in mechanisms, MI and MO are often used for motor learning and rehabilitation. For example, dancers viewing videos and mentally rehearsing their moves to learn their performance or stroke patients observing their therapists’ demonstrations and mentally rehearsing their skills during rehabilitation. Despite the similarities in mechanisms, the level of activation differs between ME, MO, and MI, and the learning trajectory might be different when using different types of methods^10,11^. Investigating the simultaneous neural activity of ME, MO, and MI, provides an understanding of the neurological correlate indexing of overt and covert action processing (i.e., movement execution vs. movement perception and/or imagery)^12^, which can be relevant for application of strategies in the context of motor learning and rehabilitation. In the current study, we used a multimodal fNIRS and EEG system to further explore the similarities and differences in neural activity during ME, MO, and MI.

The AON is suggested to be the common neural network recruited during ME, MO, and MI^13–15^. In humans, neuroimaging research has identified premotor cortex (PMC), supplementary and pre-supplementary motor areas (SMA, pre-SMA), the primary motor cortex (M1), and the inferior and superior parietal lobule (IPL, SPL) as part of the AON^16,17^. M1 was reported to be important in generating movement outputs^18^. It received processed sensory information from the parietal regions (IPS, SPL) and works in association with the PMC, SMA/pre-SMA to plan for voluntary movements^19,20^. Moreover, the parietal regions are important in planning the kinematic aspects of the movements based on the internal movement representations^21,22^. Studies investigating the AON activity have primarily used functional Magnetic Resonance Imaging (fMRI) and EEG^12,16,23,24^. Although these fMRI and EEG studies provide insights into AON correlates, technical issues prevent them from assessing activation during real-time, face-to-face ME and MO, or from localizing the areas of cortical activation elicited by these tasks^17^. For example, fMRI is constrained to a scanning bore and is sensitive to movement artifacts, making it difficult to perform naturalistic ME tasks. As a result, few fMRI studies included a real-time, ME condition^3,16,25^. Because EEG is less sensitive to motion artifacts than fMRI, it can better accommodate execution conditions; however, this modality has poor spatial specificity necessary to target specific AON regions^14,25,26^.fNIRS is a suitable alternative as it is less sensitive to motion artifacts than fMRI and has a better spatial resolution than EEG^25^. fNIRS is an optical neuroimaging technique that assesses hemodynamic changes in the cortex, measuring similar signals as fMRI (blood-oxygen dependent level, BOLD), but it is portable and fits on participants’ heads without constraint to a scanning bore^27^. Due to its low sensitivity for motion artifact, fNIRS allows for a ME condition; previous fNIRS studies have also reported AON activity similar to that detected using fMRI^14^. However, one of the disadvantages of fNIRS compared to EEG is its lower temporal resolution. Due to the timing of the hemodynamic response, fNIRS measures neural activation on the order of seconds, whereas EEG can measure electrical neural responses within milliseconds. Given the advantages of fNIRS and EEG respectively, simultaneous collection of these modalities would help characterize the AON.

Here, we propose using combined EEG-fNIRS to study ME, MO, and MI. These conditions were part of a live-action paradigm, where the participants and experimenters performed actions in front of one another. The multimodal method allows important facets of brain activity typically collected individually to be examined simultaneously. Canonical Correlation Analysis (CCA) is a classic way to evaluate the multivariate associations between two types of high-dimensional data using canonical vectors or matrices (e.g., different neuroimaging modalities)^28^. Here, we used structured sparse multiset CCA (ssmCCA) to investigate MI activity. ssmCCA is an analytic approach that was previously used on only the ME and MO conditions in this dataset^7^. Using ssmCCA, neural electrical and hemodynamic responses were fused to pinpoint the brain regions that are consistently detected by fNIRS and EEG. The Simulation Hypothesis posits that the primary motor cortex is activated during ME but not MI, as imagery activates secondary/higher-order motor regions without activating the primary motor cortex^3,29^. For this reason, we expect that the AON network will be active during MI, ME, and MO, but primary motor regions will show greater activity during ME. This study aims to elucidate the differences between ME, MO, and MI during an ecologically valid EEG-fNIRS paradigm, better characterizing “target neural mechanisms” that may benefit future therapies or interventions^30^.

## Methods

### Participants

Sixty healthy adult participants between 18 to 65 years of age were recruited at the National Institute of Health (NIH, n = 40) and the University of Maryland (UMD, n = 20). Participants with a concussion history within 12 months were excluded from study participation. Moreover, we only included participants who had > 50% of data from both EEG and fNIRS available in our analyses. Due to the challenges of simultaneously collecting high-quality EEG and fNIRS data, only 21 participants (Age (mean ± SE) = 33.1 ± 2.8) were included in the analysis. Among the 21 participants, 16 of them were right-handed, while the other 5 participants were Ambidextrous. The study protocol was approved by the National Institutes of Health and University of Maryland Institutional Review Board (NIH-IRB and UMD-IRB). All experiments were performed according to the NIH IRB/UMD IRB-approved study protocol and according to the Declaration of Helsinki. All participants signed informed consent forms before the start of the experiment, including consent to use their pictures for publication.

### Measures

A 24-channel continuous-wave functional NIRS system (Hitachi ETG-4100) was used to measure the changes in oxygenated hemoglobin (HbO) and deoxyhemoglobin (HbR) concentration. Two wavelengths in the near infrared region (695 nm and 830 nm) were measured at a sampling rate of 10 Hz. The bilateral fNIRS probe contained 8 sources and 10 detectors to form 24 channels. To index AON hemodynamic activity, optodes were placed over sensorimotor and parietal cortices^31,32^. The fNIRS probe was embedded within an elastic, 128-electrode EEG cap (Electrical Geodesic, Inc., Eugene, OR). EEG cap size was selected using each participant’s measured head circumference. As cap sizing varied by participant’s head circumference, variation in cap placement led to slight differences in inter-optode spacing (M = 2.88 ± 0.13 cm, range 2.16–3.26). Upon experiment completion, each participant’s fNIRS optodes were digitized to later account for any variance in cap positioning or inter-subject sizing differences. optode was digitized in reference to the nasion, inion, and preauricular landmarks using a 3D-magnetic space digitizer (Fastrak, Polhemus). Additional information about these methods can be found in our prior publication^7,33^.

### Experimental paradigm

**Figure 1:**
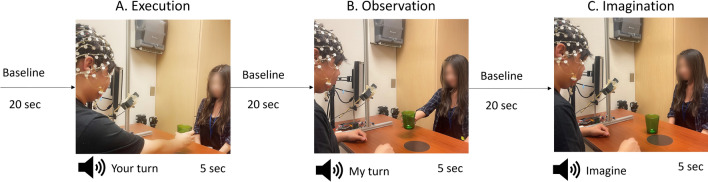
Experimental setup for each condition: (**A**) Execution, (**B**) observation, (**C**) imagination. Written permission has been taken for publication of participant pictures.

Once appropriately fitted with the fNIRS-EEG cap, the participant sat face-to-face with an experimenter across a table. Before data collection, the experimenter explained the procedure, and the participant was allowed to practice for approximately 5 min. There were three experimental conditions: (i) During ME, a pre-recorded audio command (i.e., “Your turn”) prompted the participant to grasp, lift, and move the cup approximately two feet towards themselves using their right hand (Fig. 1A). (ii) During MO, the pre-recorded audio, “my turn”, prompted the participant to watch the experimenter lift and move the cup in an identical manner (Fig. 1B). (iii) During MI, the audio announced “imagine” and the participant viewed the cup and experimenter while imagining themselves picking up and moving the cup (Fig. 1C). Cups with varied colors (i.e., green, blue, red, and transparent), sizes (i.e., small, medium, and large), and materials (i.e., plastic, and glass) were used, and their appearance was randomized across trials. Each trial lasted approximately 5 s, following a 20-s recovery period to allow for the hemodynamic response to return to baseline (Fig. 1C). Participants completed 15 trials of each condition (45 trials in total). Condition order was randomized, with no condition appearing more than three times in sequence. The experiment took approximately 45 min to complete.

### Video coding and trial exclusions

Participants’ videos were coded offline using ELAN (version 5.9) to determine the start point and the quality of each trial. For ME and MO, *start action* was coded when the experimenter/participant began moving their shoulder or arm to reach the object. For MI, the end of the audio waveform announcing the condition “Imagine” was used. Participants were only included if they completed at least five valid trials of a condition, and did not have significant movement (i.e., obvious body movements according to the video coding) or verbal artifacts (i.e., talked to the experimenter). Additional detail can be found in Debnath et al.^6^.

### Region assignments for fNIRS and EEG channels

**Figure 2:**
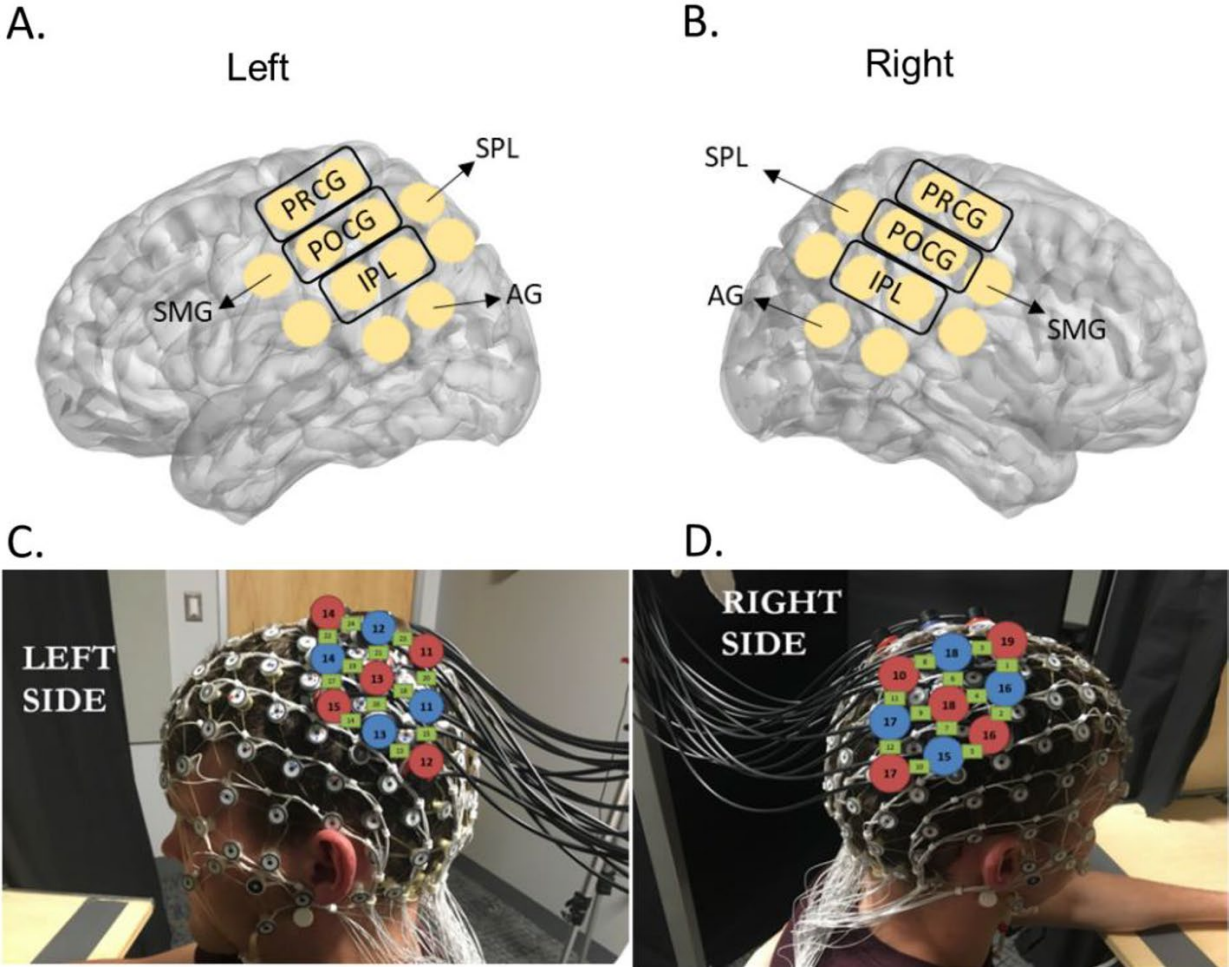
fNIRS probe placements and the ROI assignments. Written permission for publication of participant pictures has been taken.

For fNIRS channel ROI assignments, we recorded optode coordinates with the Polhemus digitizer and determined anatomical ROIs of each channel using AtlasViewer^34^. Specifically, the Polhemus digitizer was used to record the coordination of head landmarks (i.e., nasion, inion, Cz, and the tragus points of the ears) and the optodes. AltasViewer then scale the Colin brain atlas to the coordinates collected from each participant and generated the region of interest (ROI) for each channel. An ROI is included in the analyses if it was covered by at least 50% of participants (please see details in Miguel et al.^33^). The ROIs covered by the fNIRS probe set were: Precentral Gyrus (PRCG), Postcentral Gyrus (POCG), Superior Parietal Lobe (SPL), Inferior Parietal Lobe (IPL), Supra Marginal Gyrus (SMF), and Angular Gyrus (AG), located in both left and right hemispheres (please see Fig. 2A,B for the probe placements and Fig. 2C,D for the ROI assignments). For the EEG channel assignments, data from E19, E20, E23, E24, E27, E28 were combined to represent left frontal; E3, E4, E117, E118, E123, E124 for right frontal; E29, E30, E35, E36, E37, E41, E42 for left central; E87, E93, E103, E104, E105, E110, E111 for right central; E47, E51, E52, E53, E59, E60 for left parietal; E85, E86, E91, E92, E97, E98 for right parietal; E66, E69, E70, E71, E74 for left occipital; and E76, E82, E83, E84, E89 for right occipital (Supplementary Table S1).

### fNIRS unimodal data analyses

HOMER3, an open-source MATLAB software package was used to calculate the changes in the concentration of oxy-hemoglobin (HbO) and deoxy-hemoglobin (HbR) measured in micromolar (µm) (HOMER2: MGH—Martinos Center for Biomedical Imaging, Boston, MA, USA; MATLAB: The MathWorks, Inc., Natick, MA, USA). Only valid trials as assessed by behavioral coding were retained in HOMER3 for data processing. The data processing pipeline was designed according to the “Best practices for fNIRS publications”^35^. Specifically, we (1) transformed the attenuated light intensities to optical density, (2) used Principal Component Analysis (PCA, threshold set at 0.9) to remove movement artifacts and reduce physiological noises^33,36^, (3) applied automated motion detection for each channel, (4) corrected the detected motion artifacts using the spline interpolation and Savitzky-Golay filtering methods^37^, (5) low-pass filtered at 0.50 Hz to remove high frequency noise such as cardiac signal and high-pass filtered at 0.01 Hz to remove low-frequency noise such as data drift, (6) calculated the change in concentration of the hemoglobin chromophores according to the modified Beer-Lambert Law^38^, using individual Differential Pathlength Factor (DPF) values calculated using Eq. (1)^39^, (7) estimated HRF using a General Linear Model (GLM) of ordinary least squares^40^, and applied a sequence of consecutive Gaussian function to model the HRF shape^41^. The HRF time ranges were determined based on the experimental design, that is − 5 s prior and 25 s after the stimulation onset (i.e., movement initiations of the experimenter and the subjects during the Observation and Execution conditions and the end of verbal instruction during the Imagination condition). Lastly, traces were segmented into 30-s epochs, including 5 s before and 25 s after the start action (for ME and MO conditions) or the end speak (for the MI condition). In the current study, we reported both HbO and HbR findings to reduce the likelihood of false positives^42^. Z-scores were calculated for HbO and HbR separately using Eq. (2)^43^. Lastly, we conducted outlier analyses to exclude trials with Z-scores greater or less than the averaged Z-score ± 2 standard deviations. Repeated-measures ANOVA was conducted with within-group factors of condition (ME, MO, MI), and region of interest (left and right PRCG, left and right POCG, left and right SPL, left and right IPL, left and right SMG, as well as left and right AG). T-tests were used for further post-hoc analyses.

1 $$DPF\,\left(\lambda , A\right)=\alpha + \beta {A}^{\gamma }+\delta {\lambda }^{3}+\varepsilon {\lambda }^{2}+\zeta \lambda$$

where α = 223.3, β = 0.5624, A = the age of the subject, γ = 0.8493, δ = -5.723 * 10^–7^, λ = Wavelengths (695 and 830), ε = 0.001245; ζ = − 0.9025

2 $$Z\,score={(Mean}_{stim}-{Mean}_{baseline})/{SD}_{baseline}$$

### EEG unimodal analyses

EEG data were pre-processed via EEGLab software using the method proposed by Debnath and colleagues^6^. EEG channels on the boundary of the electrode net (24 channels) were excluded from analyses since they were contaminated by eye, face, and head movements (E17, E38, E43, E44, E48, E49, E113, E114, E119, E120, E121, E125, E126, E127, E128, E56, E63, E68, E73, E81, E88, E94, E99, E107). Then, high pass and low pass filters with cut-off frequencies of 0.3 and 49 Hz were applied to the continuous data. Using EEGLAB plugin FASTER artifactual channels were identified and subsequently removed^44^. In order to remove noises from eye blinks, respiration, and muscle movement, we applied extended infomax ICA to the data. Using the ADJUST plugin to EEGLAB, artifactual ICs were removed and the remaining data were epoched to − 1000 ms to 1000 ms relative to the “Start Action” marker in ME and MO conditions and “End Speak” in MI condition^45^. We converted all the epoched data into current source density (CSD) using the CSD toolbox^46,47^. Then each epoch was convolved with Mortel wavelets to estimate its spectral power in the 8–13 Hz frequency range. Please refer to Debnath et al. for the exclusion criteria during EEG preprocessing^7^. Lastly, we conducted repeated-measures ANOVA with within-group factors of condition (ME, MO, MI) and ROI (left and right frontal, left and right central, left and right parietal, as well as left and right occipital). T-tests were used for further post-hoc analyses.

### EEG-fNIRS data fusion

We applied the ssmCCA technique to integrate EEG and fNIRS datasets for multiple subjects^7^. To extract features within each dataset, we calculated the cross-modality covariations and decompose each set into a set of components, corresponding to the ROIs identified for fNIRS. Our EEG matrix for each participant consisted of the changes in the power over the α frequency band in EEG channels. The fNIRS matrix includes changes in brain oxygenated hemoglobin captured from 24 channels. We fed multimodal multi-participant data to ssmCCA with the goal of letting the modalities fully interact in a symmetric manner. Using ssmCCA, we found which brain regions given by fNIRS data have the highest correlation that is associated with the α desynchronization seen in EEG data. The proposed method is an unsupervised learning process that finds the canonical variates with or without prior information. The features are extracted by the model and predict the true covariation in different brain regions of various participants across the two modalities. As it has been shown^48,49^, the LASSO penalty allowed us to impose the sparsity to the canonical variates for EEG datasets and fused LASSO to impose both sparsity and smoothness (therefore considering spatial information) in the case of fNIRS datasets. The reason behind it is that in EEG data we only need α desynchronization information while in fNIRS data we also use structural information as well as AON activities. We can get multiple (α desynchronization, brain region) pairs; however, we only consider the first pair indicating the set with the highest correlation. These pairs were then averaged across individuals and compared between the ME, MO, and MI conditions to determine which regions were consistently active across the three conditions versus condition-specific using the ssmCCA approach. The EEG-fNIRS data combination pipeline was summarized in Supplementary Fig. S1.

## Results

### fNIRS unimodal results

**Figure 3:**
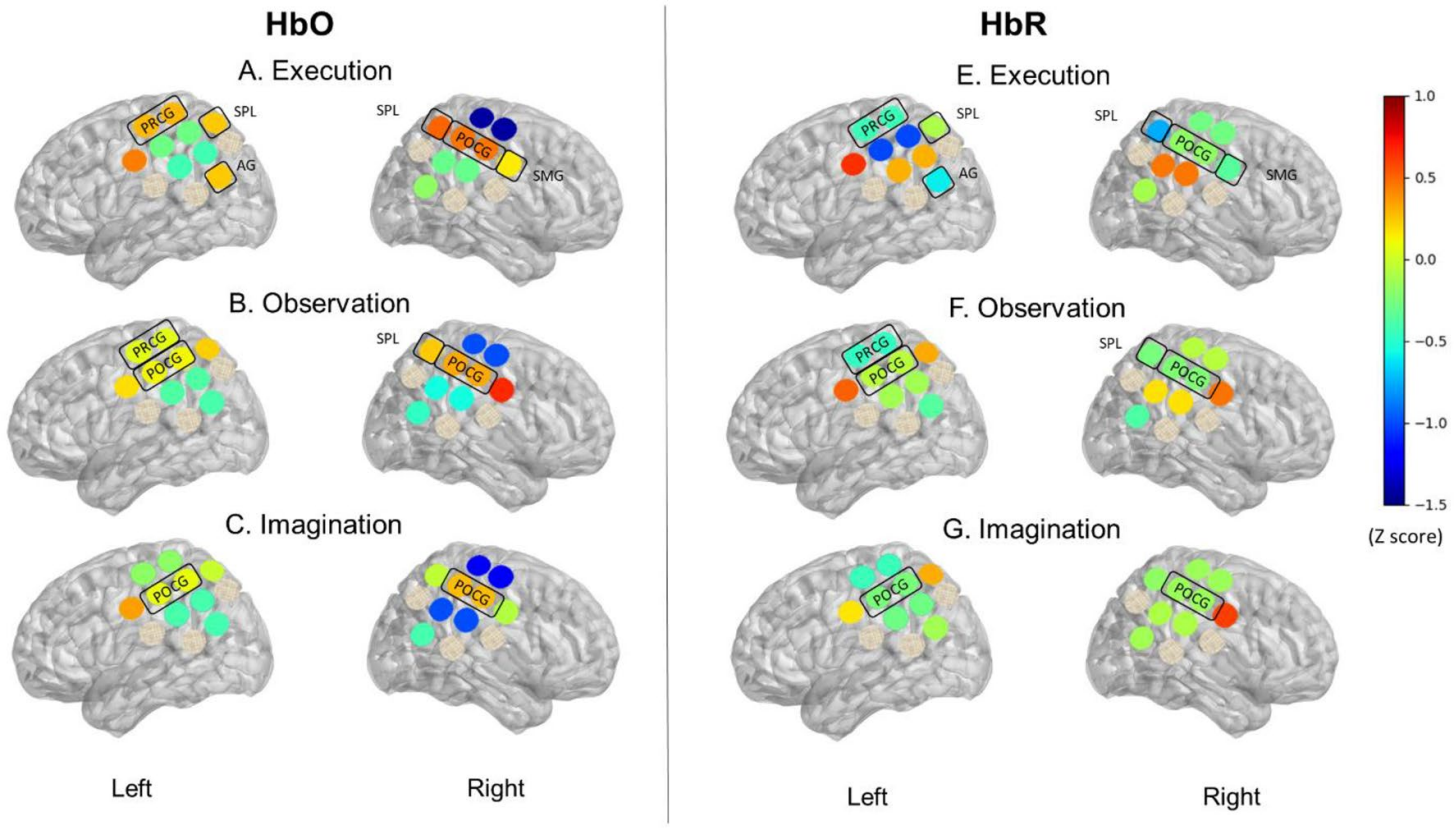
Unimodal fNIRS results. This is a visual representation of the HbO and HbR Z-scores during Execution (ME), Observation (MO), and Imagination (MI). HbO values on Y-axis range from − 1.5 indicated by cooler color (blue) to 0.1 indicated by warmer color (red). Highlighted regions indicate an increase in HbO and a decrease in HbR Z-scores. During ME, positive Z-scores for HbO and negative Z-scores for HbR were found in left PRCG, SPL, AG, and right POCG, SPL, SMG (**A**,**E**). During MO, positive HbO and negative HbR were found over Left PRCG, POCG, and right POCG, SPL (**B**,**F**). During MI, positive HbO and negative HbR were found in left and right POCG (**C**,**G**).

The Mean and Standard Error (SE) of the Z-scores for HbO and HbR were summarized in Supplementary Table S2. Figure 3 show the color-coded averaged Z-scores for HbO and HbR during ME, MO, and MI conditions. During ME, positive Z-scores for HbO and negative Z-scores for HbR were found in the left PRCG, SPL, AG, and right POCG, SPL, SMG (Fig. 3A,E). During MO, positive HbO and negative HbR were found over the left PRCG, POCG, and the right POCG, SPL (Fig. 3B,F). Lastly, during MI, positive HbO and negative HbR were found in the left and right POCG (Fig. 3C,G).

### Conditional and hemispheric differences

For HbO Z-scores, repeated-measures ANOVA revealed a significant main effect of Region (F (11, 77) = 2.412, *p* = 0.412) and a 2-way interaction of Condition × Region (F (22, 154) = 1.744, *p* = 0.027). Post-hoc analyses showed significantly greater left AG activation during ME, compared to the MO and MI conditions (Left AG, ME vs MO: t (237) = − 2.138,* p* = 0.034; left AG, ME vs MI: t (243) = 2.243,* p* = 0.026), with no significant differences between MO and MI (*p* > 0.05; Fig. 3). On the other hand, greater right SMG activation was found during MO compared to ME and MI (left SMG, MO vs ME: t (221) = 1.746, *p* = 0.082; left SMG, MO vs MI: t (221) = 2.415, *p* = 0.017), and greater left POCG were found during MO compared to ME (Left POCG, MO vs ME: t (247) = 2.044, *p* = 0.042). Lastly, lower left AG, right SPL, and right IPL activation was found during MI compared to ME (right SPL, ME vs MI: t (218) = 1.924, *p* = 0.056; right IPL, ME vs MI: t (263) = 2.125, *p* = 0.035, Fig. 3). Please see detailed t-statistics and effect sizes in Supplementary Table S3. For the HbR Z-scores, repeated-measures ANOVA revealed a significant main effect of Region (F (11, 220) = 2.184, *p* = 0.016). No significant interaction was found for HHb Z scores.

### EEG unimodal results

**Figure 4:**
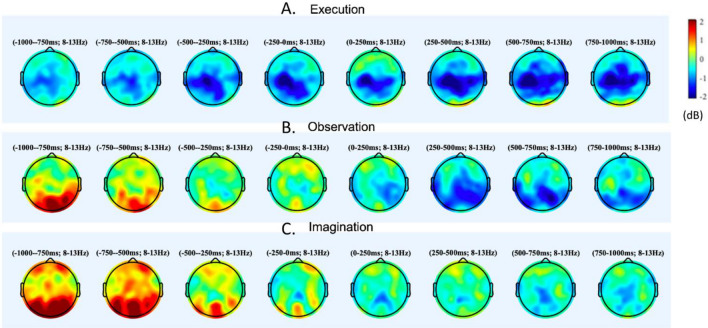
Unimodal EEG results. This is a visual representation for power synchronization. Synchronization is indicated by warmer colors (red), while desynchronization is indicated by cooler colors (blue). Significantly greater left AG activation were found during ME, compared to the MO and MI conditions (*p*s < 0.05); Greater right SMG activation was found during MO compared to ME and MI (*p*s < 0.05), and greater left POCG were found during MO compared to ME (*ps* < 0.05); Lower left AG, right SPL, IPL activation was found during MI compared to ME (*p*s < 0.05).

The repeated-measures ANOVA showed a significant main effect of Region (F (7.0, 119.0) = 2.7, *p* < 0.05) and a 2-way interaction of Condition x Region (F (14.0, 238.0) = 4.8, *p* < 0.01). Figure 4 shows the color-coded α desynchronization during ME, MO, and MI conditions. Post-hoc analyses showed the greatest desynchronization over left central region during ME compared to MO and MI (left central, ME vs MO: t (17) = − 2.50,* p* = 0.02; left central, ME vs MI: t (17) = − 2.66,* p* = 0.02). On the other hand, ME and MO elicited higher desynchronization over the right central and parietal regions compared to MI (right central, MI vs ME: t (18) = 2.40,* p* = 0.03; right central, MI vs MO: t (19) = 2.14,* p* = 0.05; right parietal, MI vs ME: t (18) = 2.14,* p* = 0.05; right parietal, MI vs MO: t (19) = 3.34,* p* = 0.03). Moreover, higher desynchronization was found over right frontal regions during MI compared to MO (t (19) = 2.68,* p* = 0.02). Please see detailed t-statistics and effect sizes in Supplementary Table S4.

### EEG-fNIRS fusion findings

**Figure 5:**
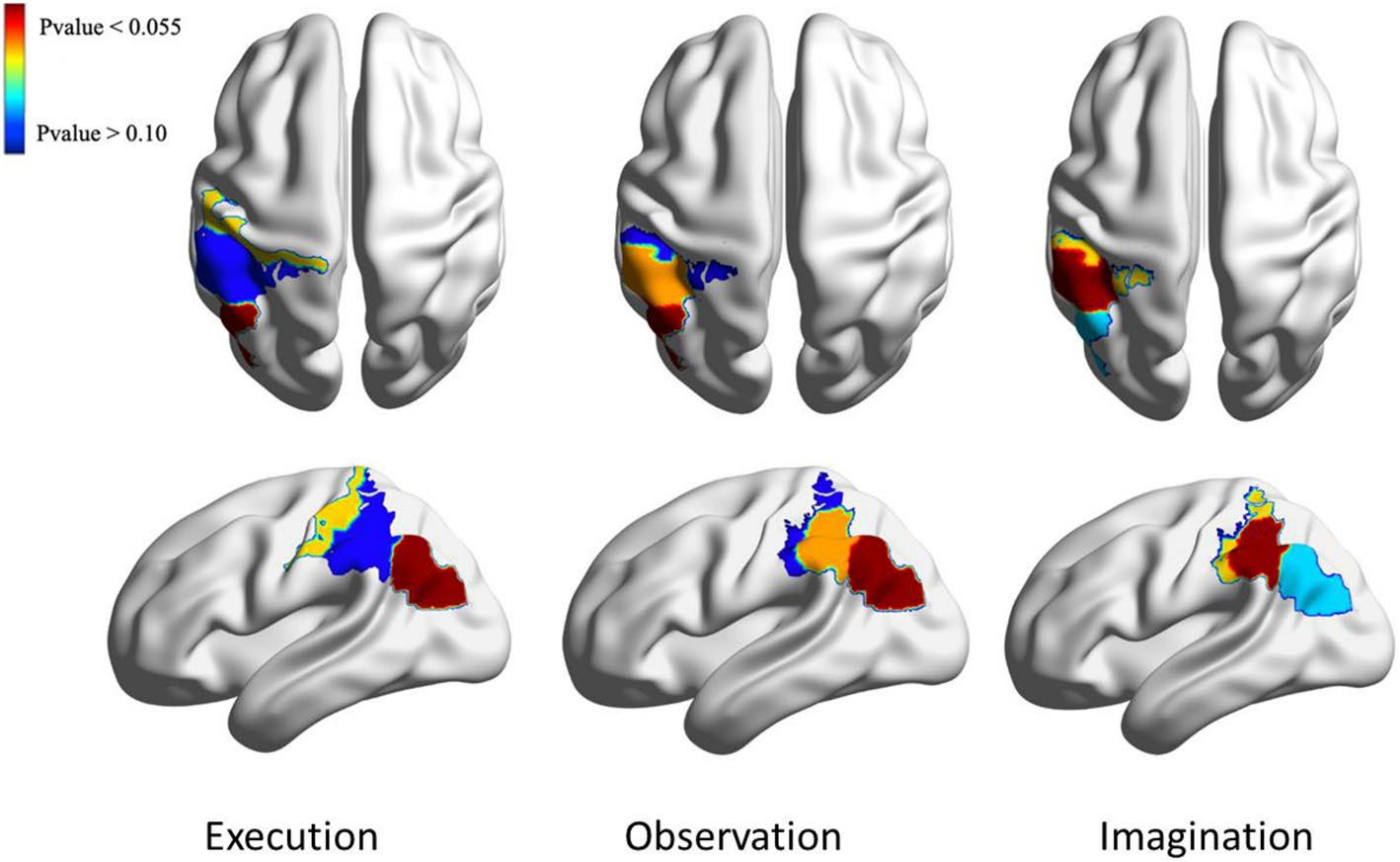
Extracted brain regions associated with execution, observation, and imagination. The axial view is on the top right, while the sagittal view is on the bottom right. The color bar refers to the *p*-values of the correlations in the region. Moderate correlations were found over the left IPL (r = 0.39; *p* = 0.059), left POCG (r = 0.38, *p* = 0.061), and left SMG (r = 0.29, p = 0.069) during ME; over the left IPL (r = 0.46; *p* = 0.053), left SMG (r = 0.38; *p* = 0.059), and left POCG (r = 0.32; *p* = 0.066) during MO; and over the left SMG (r = 0.50, *p* = 0.059), left POCG (r = 0.41; *p* = 0.061), and left IPL (r = 0.20, *p* = 0.063) during MI.

Using ssmCCA analyses, we found a set of brain regions in the left hemisphere that showed borderline significant (*p* < 0.10) correlations between the HbO changes and α desynchronization during the ME, MO, and MI conditions. Specifically, moderate correlations were found over the left IPL (r = 0.39; *p* = 0.059), left POCG (r = 0.38, *p* = 0.061), and left SMG (r = 0.29, p = 0.069) during ME; over the left IPL (r = 0.46; *p* = 0.053), left SMG (r = 0.38; *p* = 0.059), and left POCG (r = 0.32; *p* = 0.066) during MO; and over the left SMG (r = 0.50, *p* = 0.059), left POCG (r = 0.41; *p* = 0.061), and left IPL (r = 0.20, *p* = 0.063) during MI. Table 1 and Fig. 5 include cross-modality correlations and associated *p*-values, as well as the color-coded results.

**Table 1:** Cross-modality correlations and associated *p*-values during ME, MO, and MI.

	Execution			Observation			Imagination		
	Correlation	Corresponding fNIRS region	P-value	Correlation	Corresponding fNIRS region	P-value	Correlation	Corresponding fNIRS region	P-value
Comp 1	0.391	*Left IPL*	0.059	0.455	*Left IPL*	0.053	0.4966	*Left SMG*	0.059
Comp 2	0.317	*Left POCG*	0.061	0.382	*Left SMG*	0.059	0.4070	*Left POCG*	0.061
Comp 3	0.294	Left SMG	0.069	0.316	Left POCG	0.066	0.2017	Left IPL	0.063

## Discussion

To our knowledge, this is the first study that investigates neural activity during live ME, MO, and MI using simultaneous recording of fNIRS and EEG. Moreover, in addition to the unimodal analyses of fNIRS and EEG data across these conditions, we performed novel data fusion analyses to integrate the complementary information provided by the two modalities. The unimodal fNIRS analyses showed the greatest left AG activation during ME compared to MO and MI, the greatest right SMG activation during MO compared to ME and MI, as well as the lowest left AG, right SPL, and right IPL activation during MI compared to ME and MO. The unimodal EEG analyses showed the greatest α desynchronization in the left central region (i.e., μ desynchronization) during ME compared to MI and MO, while MI generated the lowest α desynchronization over the right frontal, central, and parietal regions compared to the other two conditions. The findings from the unimodal EEG and fNIRS analyses suggested similar neural mechanisms; however, the regions showing differentiated activation across the conditions were not perfectly consistent across the neuroimaging modalities. Using integrated EEG-fNIRS analyses, we determined the regions associated with AON over the left IPL, POCG, and SMG as they showed significant correlations of neural activity between the two modalities (correlation = 0.20 to 0.50). The multimodal analyses could be used to explain the discrepancies between the unimodal findings.

Though fNIRS is likely the best method for measuring brain activity during real-time action-observation tasks, there are relatively few studies that have employed this method. Using fMRI, studies have found overlapping cortical activation during ME, MO, and MI, including PMC, SMA, pre-SMA, M1, as well as IPL, SPL as part of the AON^16,17^. Our unimodal fNIRS findings are consistent with previous fMRI findings as we showed positive HbO and negative HbR Z-scores over left PRCG, SPL, AG, and right POCG, SPL, SMG during ME, over left PRCG, POCG, and right POCG, SPL during MO, and over left and right POCG during MI.

Only a few fNIRS studies compared the differences between conditions^50,51^. For example, An et al. found that although the primary sensory-motor cortex, premotor cortex, and prefrontal cortex are consistently active during ME, MO, and MI; MO elicits the lowest level of cortical activation compared to the other two conditions^50^. Likewise, Balconi et al. found similar activations in the supplementary motor area during ME and MI, but lower activation over posterior parietal regions during MI compared to ME and MO^5,52^. In our previous publication, we extensively discussed the different cortical activation between ME and MO^7^. In the current study, we added the MI condition and compared its differences in cortical activation with the other two conditions. Specifically, we found the lowest left AG and right SPL and IPL activation during MI compared to the other two conditions. The AG and parietal regions (SPL, IPL) are part of the AON and are known to be active during MO and ME^53–55^. The AG and parietal regions are important for processing sensory information and linking limb movements to internal movement representations^20,21^. MI may involve motor planning for the kinematic components (leading to high cortical activation over central regions) but less consistently rely on the AG parietal regions to refer to the sensory information and match with the internal movement representation (leading to lower cortical activation over the posterior parietal lobe).

The EEG unimodal findings suggested similar neural mechanisms; however, the regions showing conditional differences are slightly different from the fNIRS findings. For example, studies investigating the activation patterns using EEG have found overlapping neural activity during the execution, observation, and imagination of motor tasks^56^. However, a stronger α desynchronization over the central regions (also called μ desynchronization) is frequently reported during ME compared to MI and MO^57^. For the differences between ME and MO, Eaves et al. found that during movements that combine observation and imagination, there was greater α desynchronization in the sensorimotor cortex and posterior parietal regions compared to pure observation or pure imagination conditions^58^. According to the authors, pure imagination results in enhanced α desynchronization than pure observation in the sensorimotor cortex, but not in the posterior parietal regions^58^. In the current study, we also found the greatest α desynchronization over the left central region during ME compared to the other two conditions. Compared to ME and MO, we found the lowest α desynchronization over the right frontal, central, and parietal regions. The results are consistent with the previous EEG literature but are slightly different from our fNIRS unimodal findings. Specifically, we found more widespread MI-related reductions in cortical activation over the right hemisphere using EEG (i.e., right frontal, central, and parietal) compared to fNIRS (i.e., AG). These discrepancies might be because of the better coverage but lower spatial resolution in EEG than fNIRS, leading to less accurate regional findings.

Multimodal analysis of the brain gives us a more comprehensive view of brain functionality by considering relevant complementary information, potentially providing a more accurate picture when used in clinical settings. However, there are challenges associated with this goal. Not only is the nature of data from various modalities inherently different, but also their dimensionality and resolutions differ. We addressed both challenges by defining a set of multivariate features such that most of the variability in the data is still preserved. These features can establish a dimension of coherence that creates a link among the multimodal data through a common dimension. To extract features within the datasets, we calculated the cross-modality covariations and decompose each set into a set of components, corresponding to spatial areas for fNIRS. Using these components, we identified the differentially activated brain regions and the relationship between them. In simpler terms, this considers the spatial, hemodynamic, and electrical responses of the brain during our action execution, observation, and imagination paradigm. The resulting matrices from combined features of EEG and fNIRS datasets lead us to obtain brain regions that showed good correlations between EEG and fNIRS, considering a decrease of power in the EEG in the α frequency band and an increase of amplitude in the fNIRS signal. Our multimodal results suggest that the left IPL, SMG, and POCG are commonly activated during all three conditions, confirming concise regions associated with AON. Unimodal findings outside of these areas might be related to noise or other neural activity (i.e., response inhibition, decision-making, etc.) and need to be validated.

Despite the strengths of the multimodal analysis, some drawbacks of this method are worth mentioning. We included healthy participants with a relatively wide age range (18–65 years of age). While the sample size limited our ability to conduct subgroup analyses for different age groups, future longitudinal/cross-sections studies should investigate how age might play a factor to influence neural activity. The integration of distinct imaging techniques is challenging during data collection and analyses and results in higher rates of data exclusion. For example, adding the fNIRS probe holder to the EEG cap might create gaping between the scalp of the fNIRS probes and EEG sensors, leading to greater noise. Future studies would benefit from including more participants and upgrading the probe holder to reduce the data exclusion rate. Building upon the current findings, future studies can explore the use of individual/combined neuroimaging techniques in different age groups and in populations with different neurodevelopmental disorders. The use of unimodal and multimodal neuroimaging tools can be used to study infants and toddlers with or without elevated risks for developing developmental disorders, with the aim to identify the typical/atypical developmental trajectories of the AON.

## Supplementary Information

Supplementary Information.

## Data Availability

The datasets generated during and/or analyzed during the current study are available from the corresponding author on reasonable request.
