# Supplementary material for: Simultaneous multimodal fNIRS-EEG recordings reveal new insights in neural activity during motor execution, observation, and imagery
Source: Sci Rep. 2023 Mar 29;13:5151. doi: 10.1038/s41598-023-31609-5 (PMC10060581; doi:10.1038/s41598-023-31609-5)

Simultaneous Multimodal fNIRS-EEG Recordings Reveal New Insights in Neural Activity During Motor Execution, Observation, and Imagery

Supplementary Information

Supplementary Table S1. EEG Channel Assignments

ROI	Channels in Left Hemisphere	Channels in Right Hemisphere
Frontal	E19, E20, E23, E24, E27, E28	E3, E4, E117, E118, E123, E124
Central	E29, E30, E35, E36, E37, E41, E42	E87, E93, E103, E104, E105, E110, E111
Parietal	E47, E51, E52, E53, E59, E60	E85, E86, E91, E92, E97, E98
Occipital	E66, E69, E70, E71, E74	E76, E82, E83, E84, E89

Supplementary Table S2. Mean and SE for HbO and HbR Z scores

	**HbO Z score**			**HbR Z score**		
	**ME**	**MO**	**MI**	**ME**	**MO**	**MI**
**Left Hemisphere**						
PRCG	0.28 ± 0.02	0.07 ± 0.00	-0.19 ± 0.01	-0.44 ± 0.03	-0.47 ± 0.03	-0.44 ± 0.03
POCG	-0.28 ± 0.02	0.11 ± 0.01	0.09 ± 0.01	-1.01 ± 0.06	-0.06 ± 0.00	-0.24 ± 0.01
SPL	0.23 ± 0.02	0.22 ± 0.02	-0.02 ± 0.00	-0.10 ± 0.01	0.32 ± 0.02	0.31 ± 0.02
IPL	-0.42 ± 0.03	-0.38 ± 0.02	-0.41 ± 0.02	0.30 ± 0.02	-0.13 ± 0.01	-0.28 ± 0.02
SMG	0.44 ± 0.03	0.18 ± 0.01	0.35 ± 0.02	0.65 ± 0.04	0.51 ± 0.03	0.16 ± 0.01
AG	0.23 ± 0.01	-0.40 ± 0.03	-0.41 ± 0.03	-0.60 ± 0.04	-0.36 ± 0.02	-0.13 ± 0.01
**Right Hemisphere**						
PRCG	-1.43 ± 0.09	-0.99 ± 0.06	-1.23 ± 0.08	-0.27 ± 0.02	-0.07 ± 0.00	-0.14 ± 0.01
POCG	0.46 ± 0.03	0.32 ± 0.02	0.27 ± 0.02	-0.19 ± 0.01	-0.23 ± 0.01	-0.15 ± 0.01
SPL	0.50 ± 0.03	0.24 ± 0.02	-0.04 ± 0.00	-0.76 ± 0.05	-0.25 ± 0.02	-0.19 ± 0.01
IPL	-0.30 ± 0.02	-0.56 ± 0.03	-0.99 ± 0.06	0.46 ± 0.03	0.18 ± 0.01	-0.10 ± 0.01
SMG	0.14 ± 0.01	0.66 ± 0.04	-0.08 ± 0.01	-0.35 ± 0.02	0.46 ± 0.03	0.61 ± 0.04
AG	-0.19 ± 0.01	-0.47 ± 0.03	-0.42 ± 0.03	-0.11 ± 0.01	-0.38 ± 0.02	-0.13 ± 0.01

Orange shade indicates positive value, while blue shade indicates negative value.

Supplementary Table S3. T-Statistics and Effect Sizes for fNIRS Unimodal Post hoc Analyses

**Comparisons**	**t**	**df**	**p-value**	**Cohen's d (95 % CI)**
Left POCG, MO vs ME	2.04	247	0.04	0.13 (0.01 to 0.26)
Left AG, MO vs ME	-2.14	237	0.03	-0.14 (-0.27 to -0.11)
Right SMG, MO vs ME	1.75	221	0.08	0.12 (-0.02 to 0.25)
Right SMG, MO vs MI	2.42	221	0.02	-0.16 (-0.03 to 0.29)
Left AG, ME vs MI	2.24	243	0.03	0.14 (0.17 to 0.27)
Right SPL, ME vs MI	1.92	218	0.06	0.13 (-0.00 to 0.26)
Right IPL, ME vs MI	2.13	263	0.04	0.13 (0.10 to 0.25)

Supplementary Table S4. T-Statistics and Effect Sizes for EEG Unimodal Post hoc Analyses

**Comparisons**	**t**	**df**	**p-value**	**Cohen's d (95 % CI)**
Left central, ME vs MO	-2.50	17	0.02	-0.59 (-1.08 to -0.79)
Left central, ME vs MI	-2.66	17	0.02	-0.63 (-1.13 to -0.11)
Right central, MI vs ME	2.40	18	0.03	0.55 (0.06 to 1.03)
Right central, MI vs MO	2.14	19	0.05	0.48 (0.01 to 0.94)
Right parietal, MI vs ME	2.14	18	0.05	0.49 (0.01 to 0.96)
Right parietal, MI vs MO	3.34	19	<0.01	0.75 (0.24 to 1.24)
Right frontal, MI vs MO	2.68	19	0.02	0.60 (0.12 to 1.07)

Supplementary Figure S1. EEG-fNIRS data fusion pipeline

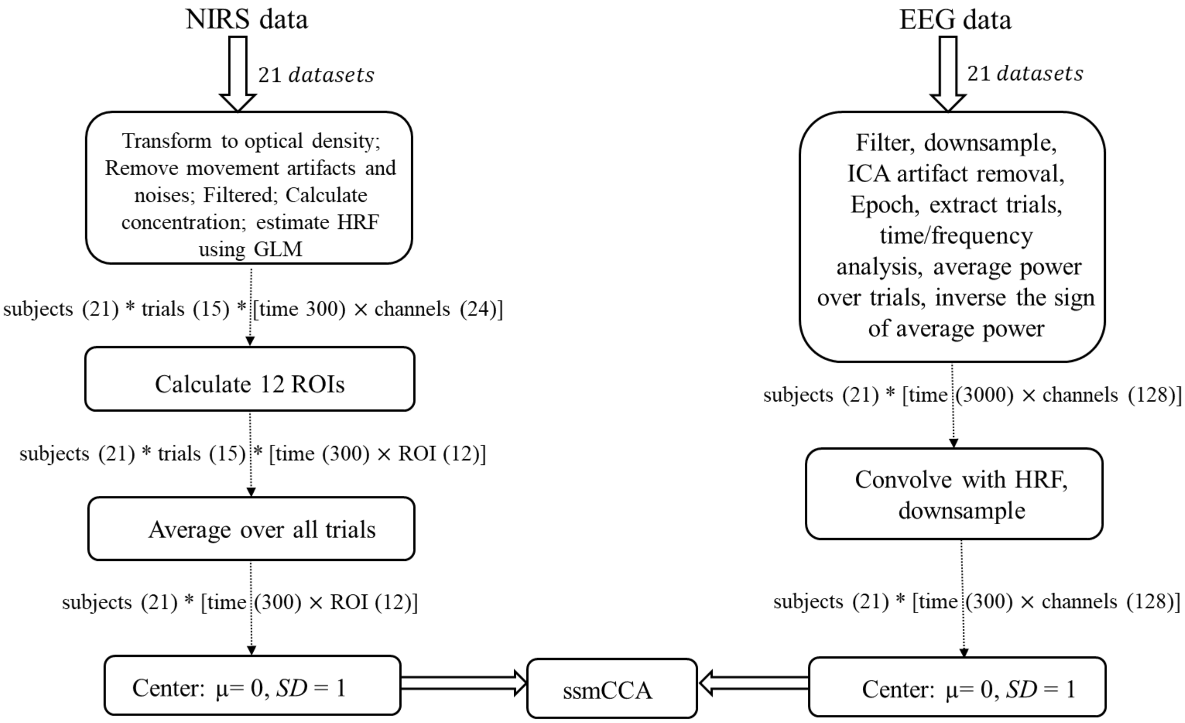

Supplement: Supplementary file 1 — Supplementary Information. [file 41598_2023_31609_MOESM1_ESM.pdf]
